# The 5′UTR variant of ERCC5 fails to influence outcomes in ovarian and lung cancer patients undergoing treatment with platinum-based drugs

**DOI:** 10.1038/srep39217

**Published:** 2016-12-14

**Authors:** Eliana Rulli, Federica Guffanti, Elisa Caiola, Monica Ganzinelli, Giovanna Damia, Marina C. Garassino, Sheila Piva, Lorenzo Ceppi, Massimo Broggini, Mirko Marabese

**Affiliations:** 1 Laboratory of Methodology For Clinical Research, Department of Oncology, IRCCS - Istituto di Ricerche Farmacologiche “Mario Negri”, 20156 Milan, Italy; 2 Laboratory of Molecular Pharmacology, Department of Oncology, IRCCS - Istituto di Ricerche Farmacologiche “Mario Negri”, 20156 Milan, Italy; 3 Oncology Department, Fondazione IRCCS Istituto Nazionale dei Tumori, 20133 Milan, Italy; 4 Oncology Department, Ospedale Fatebenefratelli e Oftalmico, 20121 Milan, Italy; 5 Clinic of Obstetrics and Gynecology, University of Milan Bicocca, San Gerardo Hospital, 20900 Monza, Italy

## Abstract

The common polymorphic variant in the 5′ untranslated region of the excision repair cross-complementation group 5 (*ERCC5*) gene was described to generate an upstream open reading frame that regulates both the basal *ERCC5* expression and its ability to be synthesized following DNA damage. This variant was reported to affect response to platinum therapy in a cohort of patients with pediatric ependymoma. The role of this variant was investigated in two cohorts of cancer patients, specifically in non-small-cell lung cancer (NSCLC) patients (N = 137) and in epithelial ovarian carcinoma (EOC) patients (N = 240), treated in first-line with platinum-based compounds. Differently from what reported for pediatric ependymoma, the analysis of the polymorphism in NSCLC patients cohort was not able to detect any difference among patients harboring different genotypes both in progression free survival (HR = 0.93; 95%CI 0.64–1.33; p-value = 0.678) and overall survival (HR = 0.90; 95%CI 0.62–1.33; p-value = 0.625). These data were corroborated in a EOC patients cohort, where similar progression free survival (HR = 0.91; 95% CI 0.67–1.24; p-value = 0.561) and overall survival (HR = 0.98; 95% CI 0.71–1.35; p-value = 0.912) were found for the different genotypes. These data, obtained in appropriately sized populations, indicate that the effect of this *ERCC5* polymorphism is likely to be relevant only in specific tumors.

Platinum-based drugs induce damages that are mainly recognized and repaired by the nucleotide excision repair (NER)^1^. Functional NER has been described to have a critical role in determining the efficacy of the platinum-based therapy in preclinical models. Cancer cells harboring a proficient NER system are more prone to repair DNA lesions and survive when treated with platinum-based compounds^2^,^3^.

*Excision repair cross-complementation group 5 (ERCC5*), also known as *Xeroderma pigmentosum group G (XPG*), is a gene belonging to NER that encodes a single-strand specific endonuclease that makes the 3′ incision in the DNA repair process following damage. Modulation of this gene was reported to affect NER activity^4^.

An increasing amount of evidence indicates that genetic variants could be important in identifying patients with a high/low probability of response to specific treatments. Genetic variants in genes belonging to the DNA repair pathways have been reported to be important determinants of platinum response^5^,^6^.

The common polymorphic variant in the *ERCC5* 5′ untranslated region (rs751402) was described to generate an upstream Open Reading Frame (uORF) that affects both the basal ERCC5 expression and its ability to be synthesized following DNA damage. This variant was reported to affect the response to platinum therapy in a cohort of patients with pediatric ependymoma. Indeed, pediatric ependymoma patients harboring the uORF (genotypes AA and AG) have a marked resistance to platinum-based therapy evaluated as a shorter progression free survival (PFS) than patients harboring the G allele^7^. The same *ERCC5* polymorphism was studied in 228 advanced chinese non-small-cell lung cancer (NSCLC) patients treated with platinum-based chemotherapy. It was shown that the AA genotype was associated with a better treatment response than the AG/GG genotypes and this was more evident in the subgroup of patients with squamous cell carcinoma^8^. In order to clarify the role of this polymorphism in affecting the response to a platinum-based therapy, the polymorphic variant in the *ERCC5* 5′ untranslated region was studied and correlated with therapeutic outcomes in two abundantly occurring malignancies, NSCLC and epithelial ovarian cancer (EOC), treated in first-line with platinum based compounds.

## Results

### NSCLC population

Between October 2007 and March 2012, patients receiving a first-line containing platinum compounds were enrolled in the TAILOR trial^9^. Of these, 137 patients were eligible for the present study. Ninety (65.7%) had GG genotype in the rs751402 locus, 43 (31.4%) harboured an AG variant, whereas four (2.9%) patients had AA polymorphism (hereafter included in the AG patients group). The minor allele prevalence was 18.6%, consistent with available data at NCBI database^10^.

For the GG population, the median age at diagnosis was 64.3 years (interquartile range (IQR): 57.3–69.9 years) whereas it was 65.5 years (IQR: 58.5–71.2 years) for the AG/AA population. The GG group was characterized by predominantly stage IIIBwet/IV (82.2%), adenocarcinoma histology (70.0%), former or never smoking habit (67.8%), ECOG-PS of 0 (58.9%) and a wild-type status of *KRAS* (75.5%). Similarly, the AG/AA patients were predominantly high stage (80.8%), with adenocarcinoma histology (70.2%), former or never smoking habit (66.0%), ECOG-PS of 0 (51.1%) and a wild-type status of *KRAS* (80.8%). None of the characteristics considered was associated with the different genotypes present in the polymorphic site rs751402 (Table 1).

**Figure 1:**
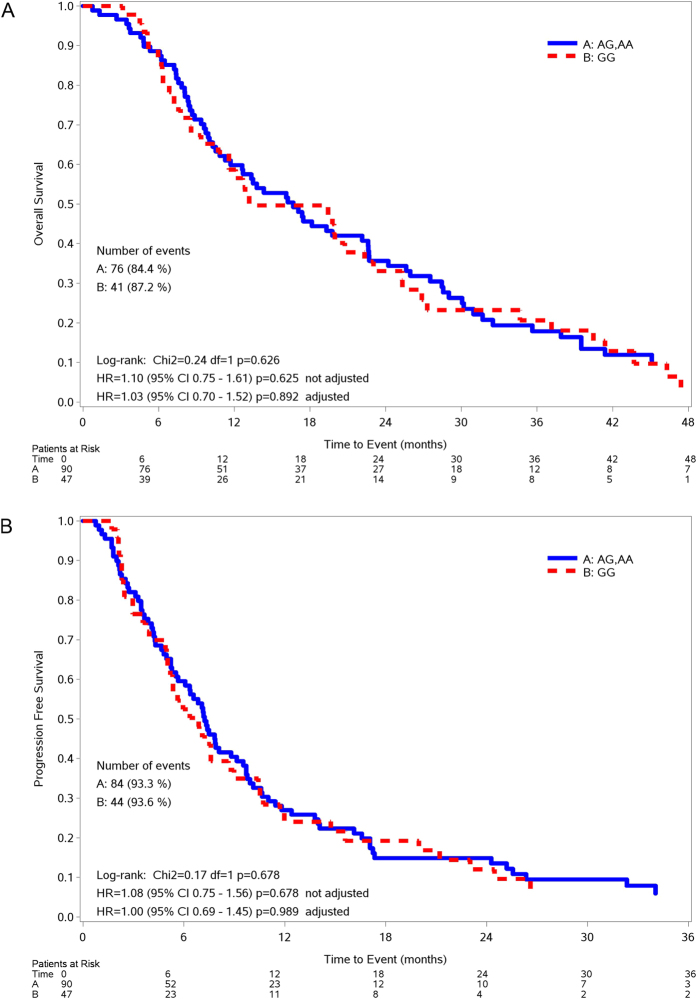
Kaplan-Meier curves for OS (**A**) and PFS (**B**) according to rs751402 genotypes in NSCLC population.

After a median follow-up of 59 months (IQR: 42–65), 128 patients progressed or died and 117 died. Median overall survival (OS) was 16.7 months (IQR 8.39–30.1 months) in the GG group and 13.2 months (IQR 7.24–27.3 months) in the AG/AA group (unadjusted hazard ratios (HR)_(GG vs AG/AA)_ = 1.10, 95% confidence intervals (CI) 0.75–1.61, p = 0.625; adjusted HR_(GG vs AG/AA)_ = 1.03, 95%CI 0.70–1.52, p = 0.892). Figure 1A shows the OS curves according to the rs751402 polymorphism. An ECOG-PS of 2 (HR = 1.56, 95%CI 1.15–2.13, p = 0.004), a IIIb wet/IV stage (HR = 1.79, 95%CI 1.09–2.03, p = 0.021) and a mutated KRAS (HR = 2.27, 95%CI 1.47–3.50, p < 0.001) were associated to a shorter OS (Table 2).

Median PFS was 7.2 months (IQR 3.88–13.8 months) in the GG group and 6.9 months (IQR 3.5–12.0 months) in the AG/AA group (unadjusted HR_(GG vs AG/AA)_ = 1.08, 95%CI 0.75–1.56, p = 0.678; adjusted HR_(GG vs AG/AA)_ = 1.00, 95%CI 0.69–1.45, p = 0.989). Figure 1B shows the PFS curves according to the rs751402 polymorphism. An ECOG-PS of 2 (HR = 1.36, 95%CI 1.03–1.80, p = 0.030), a IIIbwet/IV stage (HR = 1.83, 95%CI 1.13–2.97, p = 0.015) and a mutated KRAS (HR = 1.66, 95%CI 1.10–2.51, p = 0.016) were associated to a shorter PFS, while a squamous histotype (HR = 0.60, 95%CI 0.39–0.92, p  =  0.019) conferred a longer PFS (Table 2).

### EOC population

Between September 1979 and December 2004, blood samples were collected for 240 patients diagnosed for advanced (stage III/IV) EOC. Of these, 152 (66.4%) had GG genotype in the rs751402 locus, 70 (30.6%) harboured an AG variant whereas 7 (3.0%) patients had AA polymorphism (hereafter included in the AG patients group). For 11 patients we were unable to characterize the rs751402 locus. The minor allele prevalence was 18.3%, again consistent with available data at NCBI database^10^.

For the GG population the median age at diagnosis was 55.4 years (IQR: 46.8–65.8) whereas it was 51.7 years (IQR: 45.2–61.0) for the AG/AA population. The GG group was characterized by predominantly high grade (91.5%), serous histology (77.6%) and a residual tumor after surgery >10 cm (27.0%). Similarly, the AG/AA patients were predominantly high grade (90.9%), serous histology (76.6%) and with residual tumor after surgery >10 cm (27.3%). None of the characteristics considered was associated with the different genotypes present in the polymorphic site rs751402 (Table 3).

After a median follow-up of 11.3 years (IQR: 9.5–12.8), 186 patients progressed or died and 178 died.

**Figure 2:**
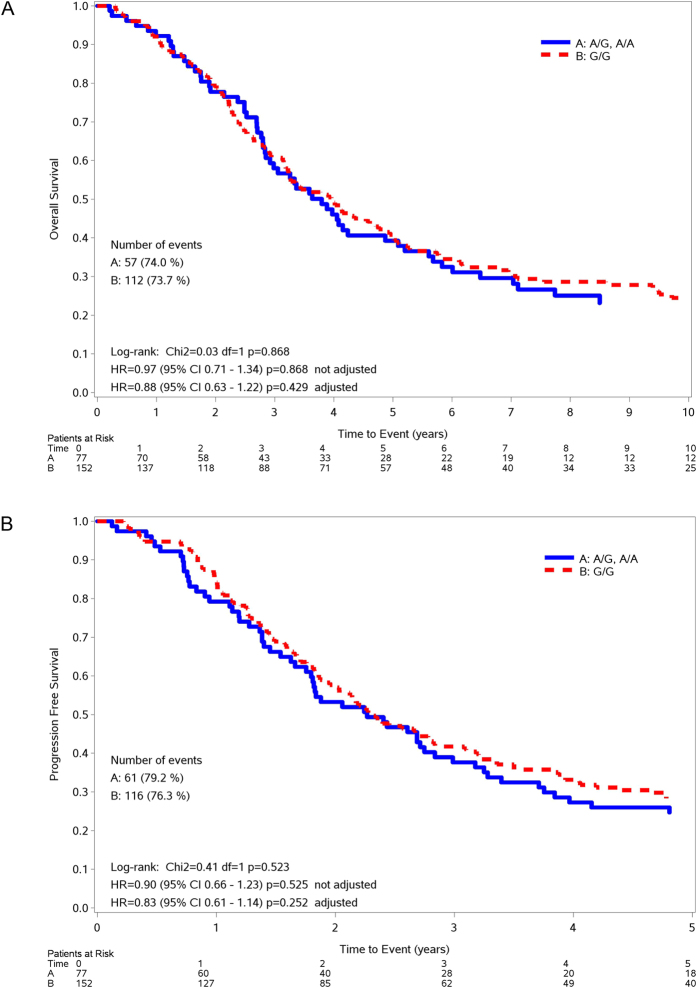
Kaplan-Meier curves for OS (**A**) and PFS (**B**) according to rs751402 genotypes in EOC population.

Median OS was 3.9 years in the GG group and 3.8 years in the AG/AA group (unadjusted HR_(GG vs AG/AA)_ = 0.97, 95%CI 0.71–1.34, p = 0.868; adjusted HR_(GG vs AG/AA)_ = 0.88, 95%CI 0.63–1.22, p = 0.429). Figure 2A shows the OS curves according to the rs751402 polymorphism. A tumor grade of 2/3 (HR = 3.69, 95%CI 1.73–7.87, p = 0.001), a residual tumor >2 cm (HR = 2.41, 95%CI 1.72–3.38, p < 0.001) and an older age (HR = 1.03, 95%CI 1.01–1.04, p < 0.001) were the characteristics associated to a shorter OS (Table 4).

Median PFS was 2.3 years in both the GG and in the AG/AA group (unadjusted HR_(GG vs AG/AA)_ = 0.90, 95%CI 0.66–1.23, p = 0.525; adjusted HR_(GG vs AG/AA)_ = 0.83, 95%CI 0.61–1.14, p = 0.252). Figure 2B shows the PFS curves according to the rs751402 polymorphism. Tumor grade of 2/3 (HR = 3.03, 95%CI 1.55–5.92, p = 0.001), residual tumor >2 cm (HR = 2.18, 95%CI 1.57–3.02, p < 0.001) and an older age (HR = 1.02, 95%CI 1.01–1.04, p < 0.001) were the characteristics associated to a shorter PFS (Table 4).

## Discussion

The identification of factors able to select patients who would better benefit from therapy is one of the big challenge in oncology. It is well known that some somatic mutations or rearrangements such as gene duplication or deletion in the tumor define subgroup of patients more responsive to chemotherapy^11^,^12^. In addition to genetic abnormalities of the tumor, the genotype of the patient could play an important role given that polymorphic variants in particular genes have been shown to influence the response to treatments^13^,^14^,^15^,^16^.

The response of cells to platinum-based compounds is strictly influenced by their ability to manage the DNA lesions induced by drug treatments^17^. The NER pathway is one of the major DNA repair pathway in mammalian cells and has been shown to have a key role in the removal of platinum-based DNA adducts^18^. Dysfunctions in NER pathway have been associated to a different sensitivity of cells to platinum-based drugs^19^ and preclinical evidence suggests that cells with high expression of NER genes are less sensitive to platinum-based compounds^20^,^21^. On the other hand, the translation of these data in the clinic setting has been more complex and conflicting data have been reported^22^,^23^,^24^.

The *ERCC5* gene, also known as *XPG*, is one of the essential DNA repair enzymes of the NER pathway^4^. XPG expression has been reported to be a determinant of platinum-based molecules activity in preclinical studies^25^. Its role as a predictive biomarker of response to platinum-based treatment in the clinic is conflicting. Indeed, while a positive correlation between XPG protein expression and response to chemotherapy was reported^26^ when the XPG mRNA levels were considered, the correlation was not found^27^.

The heterogeneity in the treatment response might be partially explained by the inter-individual genomic heterogeneity due to polymorphic sites. In this regard, the polymorphisms in the promoter region of the genes are of particular interest.

Recently, a common polymorphic variant in the *ERCC*5 5′ untranslated region was reported. Somers and colleagues proposed that the presence of the A allele in the position −420 upstream of the physiological AUG start codon generates a new open reading frame in addition to the canonical *ERCC5* ORF sited at position −177 from AUG. The presence of the A allele both in homozygosity or heterozygosity was shown to affect the basal protein expression and its ability to be synthesized following DNA damage. In fact, after DNA damage induced by cisplatin treatment, a global reduction of protein synthesis was demonstrated, but cells harboring the A allele in rs751402 site showed a maintenance of ERCC5 protein expression. The clinical corroboration of this finding was that pediatric ependymoma patients harboring the uORF (A allele), have a marked resistance to platinum-based therapy as shown by the reduced PFS as compared to patients with the G allele^7^. These data, however, contrasted with the ones reported by He and colleagues who investigated the same genetic variant in a cohort of Asiatic population (N = 228) affected by advanced NSCLC treated with platinum-based compounds. They reported that the A allele homozygous patients had a better response to cisplatin treatment than the AG and GG genotype patients^8^. It has to be stressed that this conflicting results in NSCLC have been obtained considering the AA allele different from AG while is known that AA and AG both affect ERCC5 protein expression and function^7^.

To better define the role of this polymorphic site in the response to a platinum based therapy, we studied the same polymorphism in two well characterized cohorts of cancer patients affected by two of the most common malignancies worldwide, NSCLC and EOC. The patients analyzed in this study have been treated with platinum-based chemotherapy in first-line and have long term clinical follow-up, allowing the investigation of the impact of the genomic variant on clinical outcomes. Although we analyzed a quite large number of patients, our study was unable to detect any role for the polymorphic variant in the *ERCC5* 5′ untranslated region in affecting outcomes in both the EOC and NSCLC patients. To note, the HRs obtained in both populations were very close to 1 supporting the hypothesis that the variant has no role on the prognosis of these patients. Based on presented data, obtained in appropriately sized populations, we can conclude that the effect of the *ERCC5* polymorphism in the 5′ untraslated region has no effects on response to therapy of NSCLC and EOC Caucasian patients. Peculiar type of tumor and ethnicity (considering comparable histopathological characteristics) could be responsible for the different results obtained in pediatric ependymoma and NSCLC population respectively.

## Methods

### NSCLC patients

Participating centers registered all consecutive patients with NSCLC before or during first-line chemotherapy to participate to TAILOR trial^9^. All patients received platinum-based chemotherapy in combination with either vinorelbine, gemcitabine or pemetrexed according to the physician’s choice. Patients with EGFR mutations, early stages patients and patients receiving the adjuvant therapy were excluded from this analysis. Other inclusion or exclusion criteria have been published elsewhere^9^,^28^. Research protocol was approved by the Ethics Committee of Ospedale Fatebenefratelli e Oftalmico, Milan (03 October 2007) and the study has been carried out following the principles of the Declaration of Helsinki. All patients who were eligible for participation provided written informed consent with all applicable governing regulations before undergoing any study procedure.

### EOC patients

From September 1979 to December 2004 San Gerardo Hospital (Monza, Italy) EOC patients with biological material available were consecutively enrolled in the present study. All patients received platinum-based chemotherapy as first-line. The study has been carried out following the principles of the Declaration of Helsinki and the Ethics Committee of San Gerardo Hospital, Monza, Italy approved the collection and usage of blood samples. Written informed consent was obtained from all patients before undergoing any study procedure.

### Samples collection and genotyping

Blood samples were collected in tubes containing K_2_EDTA and stored at −20 °C. DNA was extracted from blood samples using Maxwell 16 Blood DNA Purification Kit (Promega, Milan, Italy). The rs751402 polymorphism was genotyped using a TaqMan SNP Genotyping assay (Life Technologies, Monza, Italy), based on Real Time PCR technique (ABI 7900, Life Technologies). The PCR was carried out in 384-wells plates with a reaction volume of 5 μL containing TaqMan Genotyping Master Mix (Life Technologies), MGB probes and primers and 10 ng of genomic DNA. Primers and probes sequences are property of Life Technologies. Completed PCR plates were analysed using the TaqMan Genotyper Software (Life Technologies).

### Statistical methods

Baseline covariate distributions were summarised using descriptive statistics (median and interquartile range) for continuous variables; absolute frequencies and percentages for categorical variables). Wilcoxon-Mann-Whitney test for continuous covariates and Chi-square test for categorical covariates were used to detect statistical association. Progression free survival was defined as the time from the day of first-line treatment start up (NSCLC analysis) or the time from the date of diagnosis (EOC analysis) to the date of first progression or death from any cause, whichever came first. Overall survival was defined as as the time from the day of first-line treatment start (NSCLC analysis) or from the date of diagnosis (EOC analysis) to the date of death from any cause. Patients alive and without disease progression at the date of study cut-off were censored at the last available information on status. Survival curves were estimated with the Kaplan-Meier method. Cox proportional hazards models were used for univariate and multivariate (adjusted for ECOG-PS, histology and KRAS status for NSCLC analysis; adjusted for age, tumor grade and residual tumor for EOC analysis) analysis to estimate the association between polymorphism and progression free survival or overall survival. Results were expressed as hazard ratios and their 95% confidence intervals. Statistical analyses were carried out using SAS version 9.2 (SAS Institute, Cary, NC).

## Additional Information

**How to cite this article**: Rulli, E. *et al*. The 5′UTR variant of ERCC5 fails to influence outcomes in ovarian and lung cancer patients undergoing treatment with platinum-based drugs. *Sci. Rep.* **6**, 39217; doi: 10.1038/srep39217 (2016).

**Publisher’s note:** Springer Nature remains neutral with regard to jurisdictional claims in published maps and institutional affiliations.

## Figures and Tables

**Table 1:** NSCLC patients’ characteristics.

		Total	GG		AG/AA		*P-value*
			*N*	%	*N*	%	
Patients		137	90	65.7	47	34.3	
Age	Median(IQR)		64.3 (57.3–69.9)		65.5 (58.5–71.2)		0.268
Gender	Male	98	66	73.3	32	68.1	0.520
	Female	39	24	26.7	15	31.9	
ECOG-PS	0	77	53	58.9	24	51.1	0.300
	1	54	34	37.8	20	42.5	
	2	6	3	3.3	3	6.4	
Smoking	Never/ex smokers	92	61	67.8	31	66.0	0.830
	Smokers	45	29	32.2	16	34.0	
Stage at diagnosis	IIIA, IIIB	25	16	17.8	9	19.2	0.844
	IIIB wet, IV	112	74	82.2	38	80.8	
Histotype	Adenocarcinoma	96	63	70.0	33	70.2	0.868
	Squamous	34	23	25.5	11	23.4	
	Other	7	4	4.5	3	6.4	
KRAS status	Wild-type	106	68	75.5	38	80.8	0.500
	Mutated	31	22	24.5	9	9.2	

**Table 2:** Prognostic evaluation of clinical and histopathological characteristics in NSCLC population.

	Overall Survival				Progression Free Survival			
	HR	Lower 95% HR	Upper 95% HR	*P-value*	HR	Lower 95% HR	Upper 95% HR	*P-value*
Unadjusted								
rs751402 (GG vs GA/AA)	1.10	0.75	1.61	0.625	1.08	0.75	1.56	0.678
Age at diagnosis	0.97	0.63	1.47	0.872	0.85	0.57	1.26	0.413
ECOG-PS (2 vs 1 vs 0)	1.56	1.15	2.13	0.004	1.36	1.03	1.80	0.030
Histotype (squamous vs others)	0.67	0.43	1.05	0.077	0.60	0.39	0.92	0.019
Tumor stage (IIIB wet, IV vs IIIA, IIIB)	1.79	1.09	2.03	0.021	1.83	1.13	2.97	0.015
Smoking (smoking and former vs not smoking)	1.13	0.76	1.68	0.545	1.05	0.72	1.53	0.812
Sex (F vs M)	0.83	0.55	1.24	0.355	1.04	0.71	1.52	0.857
*KRAS* (mut vs wt)	2.27	1.47	3.50	<0.001	1.66	1.10	2.51	0.016
Adjusted*								
rs751402 (GG vs GA/AA)	1.03	0.70	1.52	0.892	1.00	0.69	1.45	0.989

^*^Adjusted for ECOG-PS, histology and KRAS status.

**Table 3:** EOC patients’ characteristics.

		Total	GG		AG/AA		*P-value*
			*N*	%	*N*	%	
Patients		229	152	66.4	77	33.6	
Age	Median(IQR)	54.3 (46.5–64.9)	55.4 (46.8–65.8)		51.7 (45.2–61.0)		0.084
Grade	Borderline, 1	20	13	8.5	7	9.1	0.892
	2, 3	209	139	91.5	70	90.9	
Histotype	Serous	177	118	77.6	59	76.6	0.623
	Endometrioid	22	16	10.5	6	7.8	
	Other	30	18	11.9	12	15.6	
Residual tumor	NED, micro	39	27	17.8	12	15.6	0.878
	<1 cm	29	21	13.8	8	10.4	
	1–2 cm	14	7	4.6	7	9.1	
	2–5 cm	35	20	13.2	15	19.5	
	5–10 cm	50	36	23.7	14	18.2	
	>10 cm	62	41	27.0	21	27.3	

**Table 4:** Prognostic evaluation of clinical and histopathological characteristics in EOC population.

	Overall Survival				Progression Free Survival			
	HR	Lower 95% HR	Upper 95% HR	*P-value*	HR	Lower 95% HR	Upper 95% HR	*P-value*
Unadjusted								
rs751402 (GG vs GA/AA)	0.97	0.71	1.34	0.868	0.90	0.66	1.23	0.525
Age at diagnosis	1.03	1.01	1.04	<0.001	1.02	1.01	1.04	<0.001
Residual tumor (>2 cm vs <2 cm)	2.41	1.72	3.38	<0.001	2.18	1.57	3.02	<0.001
Grade (2, 3 vs borderline, 1)	3.69	1.73	7.87	0.001	3.03	1.55	5.92	0.001
Histotype Serous ref.	1				1			
Endometrioid	1.03	0.60	1.76	0.911	1.02	0.62	1.69	0.940
Other	1.33	0.86	2.05	0.203	1.21	0.79	1.86	0.373
Adjusted*								
rs751402 (GG vs GA/AA)	0.88	0.63	1.22	0.429	0.83	0.61	1.14	0.252

^*^Adjusted for age, tumor grade and residual tumor.
